# A2ML1 Inhibits Esophageal Squamous Cell Carcinoma Progression and Serves as a Novel Prognostic Biomarker

**DOI:** 10.1155/2023/5557546

**Published:** 2023-11-03

**Authors:** Xiaoyun Zhang, Chaogui Tang, Jianchun Lian, Yuzhang Jiang

**Affiliations:** Department of Medical Laboratory, The Affiliated Huaian No. 1 People's Hospital of Nanjing Medical University, Nanjing, Jiangsu 223300, China

## Abstract

Studies have established a correlation between *α*2-macroglobulin-like 1 (A2ML1) and the prognosis of lung, pancreatic, and breast cancers; however, research on its involvement in the pathogenesis of esophageal carcinoma remains limited. Therefore, in this study, we aimed to investigate the role of A2ML1 in the progression of esophageal squamous cell carcinoma (ESCC). Immunohistochemical staining was employed to assess the expression level of A2ML1 protein in both tumor and adjacent normal tissues of patients with ESCC. The Kaplan–Meier method, along with univariate and multivariate Cox risk ratio analyses, was used to determine survival rates and prognostic factors. Furthermore, two human ESCC cell lines, KYSE30 and KYSE150, were used to assess the effect of A2ML1 overexpression on cell proliferation and apoptosis. A human apoptosis antibody kit was also used to analyze the downstream action proteins of A2ML1, and a nude mouse xenotransplantation model was used to evaluate the effect of A2ML1 on ESCC tumorigenesis *in vivo*. The protein level of A2ML1 in ESCC tissues was significantly lower than that in normal esophageal tissues, and higher A2ML1 protein levels were associated with smaller ESCC tumor sizes and improved tumor-specific survival rates. Multivariate analysis established A2ML1 as a novel independent prognostic factor for ESCC. Moreover, A2ML1 overexpression significantly inhibited ESCC cell proliferation and promoted apoptosis. A2ML1 consistently inhibited tumor growth in mouse models. Furthermore, the human apoptotic antibody kit results showed increased expression of the proliferation-inhibiting protein p21 downstream of KYSE150 cells overexpressing A2ML1. Our findings demonstrate that a correlation exists between A2ML1 and ESCC prognosis and that A2ML1 plays an antitumor role in ESCC progression. This study underscores the potential of A2ML1 as a novel biomarker for predicting the prognosis of ESCC.

## 1. Introduction

Esophageal carcinoma (EC) originates from the esophageal epithelia, extending from the lower pharynx to the esophagogastric junction. EC stands as one of the most common malignant tumors, ranking sixth as the leading cause of cancer-related deaths worldwide [1]. According to histopathological characteristics, EC is mainly divided into esophageal squamous cell carcinoma (ESCC) and esophageal adenocarcinoma. ESCC prevails in Asia and East Africa, whereas esophageal adenocarcinoma is predominant in Western countries [2, 3]. In addition, most patients with early-stage EC are asymptomatic, leading to diagnosis in the middle and late stages. Treatment modalities primarily encompass chemotherapy, radiotherapy, and targeted therapy. However, the prognosis for patients with advanced ESCC remains poor, with a five-year survival rate of less than 10% [4–6]. Therefore, an in-depth exploration of the underlying molecular mechanism of the pathogenesis of ESCC holds the potential to revolutionize diagnostic and treatment strategies to improve ESCC outcomes.

Alpha-2-macroglobulin-like 1 (A2ML1), a recently identified member of the macroglobulin inhibitor family, similar to *α*2-macroglobulin and pregnancy zone protein, is located on chromosome 12p13.31. The A2ML1 gene encodes a 1454 amino acid sequence and is a 180KD protein monomer distributed in human epithelial cells [7]. Similar to the functional characteristics of A2Ms, the A2ML1 protein can inhibit extracellular protease through a unique “capture” mechanism, thereby contributing significantly to epithelial cell desquamation. Moreover, A2ML1 can regulate epithelial cell homeostasis by interacting with growth factors and cytokines [8, 9]. Recent studies have also established a correlation between A2ML1 and the occurrence and development of otitis media, pemphigus, and Noonan syndrome [10, 11]. Moreover, A2ML1 has been associated with the prognosis of lung, pancreatic, and breast cancer [12–14]. However, there is a dearth of research on A2ML1 expression and its involvement in the pathogenesis of EC. Therefore, investigating the effect of A2ML1 on the occurrence and development of ESCC, as well as its underlying mechanisms, holds paramount importance for the identification of novel targets for ESCC diagnosis and treatment.

In this study, we aimed to assess the role of A2ML1 in ESCC and its potential underlying mechanisms. By employing immunohistochemistry (IHC), we examined the correlation between A2ML1 expression and clinical indicators and the prognosis of patients with ESCC. Subsequently, we conducted assessments on cell proliferation and apoptosis in A2ML1 overexpression and control cells to gain insights into the biological function of A2ML1 in ESCC. A human apoptosis antibody kit was used to detect the gene protein expression and explore potential molecular mechanisms. Our results revealed the molecular mechanism through which A2ML1 inhibits ESCC cell proliferation and promotes apoptosis, thus providing new insights into the role of A2ML1 in the occurrence of ESCC.

## 2. Materials and Methods

### 2.1. Patient Information and Tissue Samples

mRNA gene expression data and clinical information were obtained from The Cancer Genome Atlas (TCGA) (https://cancergenome.nih.gov/) and Genotype-Tissue Expression (GTEx) (https://gtexportal.org/home/) databases. Between December 2012 and November 2013, we collected tumors and adjacent normal esophageal tissues from 94 patients with ESCC who underwent radical resection at the Department of Thoracic Surgery, Affiliated Huaian No. 1 People's Hospital of Nanjing Medical University. All patients were pathologically diagnosed with ESCC after a preoperative endoscopic biopsy and their specimens were classified according to the tumor, node, and metastasis classification of the International Union of Cancer, the fifth edition. The overall survival curve, according to A2ML1 relative expression and cut-off value, was generated using the Kaplan–Meier method. This study was approved by the Ethics Committee of the Affiliated Huaian No. 1 People's Hospital of Nanjing Medical University (KY-2022-116-01).

### 2.2. Immunohistochemistry Analysis

A2ML1 expression in ESCC tissue samples was assessed using IHC. First, the tissue samples were subjected to dewaxing and antigen repair. Subsequently, the tissue sections were sealed in phosphate-buffered saline with Tween (PBST) buffer solution containing 1% bovine serum albumin for 30 min, followed by overnight incubation with a primary antibody (1 : 50, Abcam, UK) at 4°C. Then, the tissue sections were washed thrice and incubated with horseradish peroxidase polymer binding secondary antibody (Thermo Fisher, CA, USA). Finally, the sections were washed with PBST buffer and immunoreactivity was observed using a diaminobenzidine chromogenic agent, followed by hematoxylin staining. The sections were scanned using a pathological imaging system. Indica Labs' HALO^TM^ software was used for quantitative analysis.

### 2.3. Cell Culture

KYSE30 and KYSE150 cell lines were purchased from the Chinese Type Culture Collection (Shanghai, China). The cells were cultured in Dulbecco's Modified Eagle's Medium containing 10% fetal bovine serum and 1% penicillin/streptomycin. All cells were cultured in a humidified incubator (Thermo Scientific, USA) at 37°C and 5% CO_2_. Human A2ML1 cDNA was synthesized and inserted into pcDNA3.1 plasmids for overexpression, and cells transfected with pcDNA3.1 vector alone served as controls (OBiO Technology, Shanghai, China). Ki67 staining scores were evaluated independently by two pathologists. The proportion score was recorded in a minimum of four random fields and presented as the fraction of stained cells (0 < 10%, 1 = 10–25%, 2 = 26–75%, and 3 > 75%). The intensity score denoted the average staining intensity (0 = none, 1 = weak, 2 = intermediate, and 3 = strong). The expression of Ki67 was determined as the product of the proportion and intensity scores.

### 2.4. Cell Counting Kit-8 Assays

ESCC cells overexpressing A2ML1 were seeded into a 96-well flat plate at a density of 2000 cells per well, with 6 wells for each sample. At 24, 48, and 72 h, 10 *μ*L of Cell Counting Kit-8' (CCK-8) solution was added to each well and incubated at 37°C for 2 h. Absorbance was measured at 450 nm using an automatic microplate reader (Thermo, Shanghai, China).

### 2.5. Colony Formation Assay

Following cell counting, 500 ESCC cells were inoculated into a 6-well plate at a single cell density. Two duplicate wells were prepared for each treatment group. Fresh culture medium was replaced every 2 d, and after 10 d, the culture medium was discarded and cell colonies were fixed with polyformaldehyde and stained with 0.5% crystal violet to determine the number of stained colonies.

### 2.6. Cell Apoptosis Assays

We used the YF647-conjugated Annexin V/propidium iodide (YF647-Annexin V/PI) apoptosis detection kit (US Everbright, Beijing, China) according to the manufacturer's instructions. In brief, cells were collected via mild trypsinization, washed twice in cold PBS, stained with YF647-Annexin V and PI on ice for 10 min, and analyzed for apoptosis via flow cytometry using FACScan (Becton Dickinson, Franklin Lakes, NJ, USA).

### 2.7. Western Blotting

Cells were lysed on ice for 20 min using radioimmunoprecipitation assay' strong buffer solution with appropriate volume supplemented with protease inhibitor. After centrifugation at 4°C and 12000 rpm for 15 min, the supernatant was collected. Protein concentration was determined using a bicinchoninic acid protein determination kit (Biosharp, Shanghai, China). Equivalent amounts of proteins were separated via electrophoresis and transferred onto a polyvinylidene fluoride membrane. The membrane was sealed with 5% skim milk for 2 h and incubated overnight with a specific primary antibody. After washing thrice with TBST (10 min at each time), the membranes were incubated with a secondary antibody for 1 h and washed thrice with TBST. Finally, an enhanced chemiluminescence matrix kit (US Everbright, Beijing, China) and a ChemiDoc XRS + S system (Bio-Rad, CA, USA) were used to image the films. The following antibodies were used: anti-A2ML1 (1 : 2000, Abcam, UK), anti-glyceraldehyde 3-phosphate dehydrogenase (1 : 5000, GeneTex, USA), and goat polyclonal antibody against retinoblastoma protein (1 : 5000, ABclonal, Wuhan, China).

### 2.8. Human Apoptosis Antibody Array Assay

Relative levels of 43 apoptosis-related proteins in cell lysates were assessed using the RayBio Human Apoptosis Antibody Kit (RayBiotech, Norcross, GA, USA), following the manufacturer's instructions. Signal visualization was performed using an Axon GenePix 4000 B microarray scanner (Axon Instruments, Union City, CA, USA) and spot density was analyzed using AAH-APO-G1 software (RayBiotech).

### 2.9. Animal Experiments

Six-week-old SCID/NOD mice were purchased from the Nanjing Senbao Biological Products Center (Nanjing, China). Transfected KYSE150 cells (1 × 10^7^) were subcutaneously injected into the abdominal cavities of the mice. Tumor growth was monitored and measured weekly. The tumor volume was calculated by using the following equation:

(1) $$\begin{array}{c} V\left({\text{mm}}^{3}\right)=\frac{L\times W^{2}}{2}, \end{array}$$

where *L* = tumor length and *W* = tumor width.

On the 28th day, five mice in each group were euthanized and the tumors were harvested. The animal experiment was approved by the Animal Experimentation Ethics Committee of the Affiliated Huaian No. 1 People's Hospital of Nanjing Medical University (DW-P-2022-007-01).

### 2.10. Statistical Analysis

Data were analyzed using SPSS 19.0 software and graphs were generated using the GraphPad Prism v6 software. The correlation between A2ML1 and clinicopathological variables was assessed using the chi-square test. Survival rates and prognostic factors were determined using the Kaplan–Meier method and univariate and multivariate Cox risk ratio analyses. Data are presented as mean ± standard deviation and the mean value between groups was compared using Student's *t-*test: *P* < 0.05 (^*∗*^), *P* < 0.01 (^*∗∗*^), *P* < 0.001 (^*∗∗∗*^), and *P* < 0.0001 (^*∗∗∗∗*^) represent statistically significant differences.

## 3. Results

### 3.1. Protein Expression and Clinical Significance of A2ML1 in ESCC

**Figure 1:**
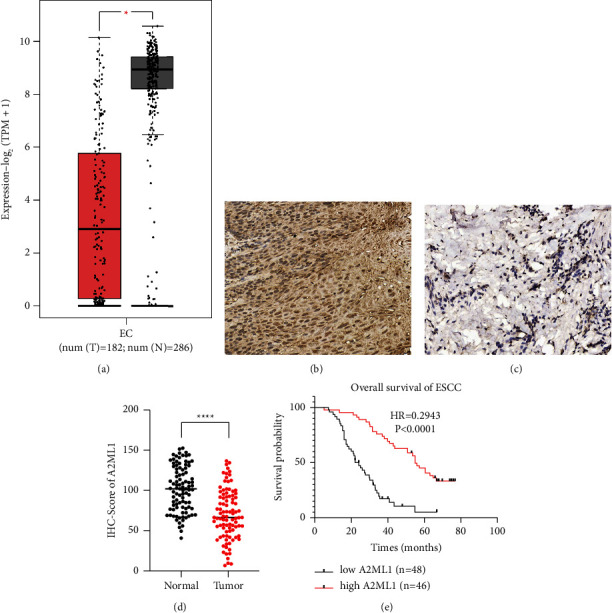
Alpha-2-macroglobulin-like 1 (A2ML1) expression was downregulated in esophageal squamous cell carcinoma (ESCC) tissues and correlated with poor patient prognosis. (a) A2ML1 mRNA levels were compared between esophageal carcinoma and normal esophageal tissues using The Cancer Genome Atlas and Genotype-Tissue Expression databases; ^*∗*^*P* < 0.05, Student's *t*-test. (b) High A2ML1 protein expression in normal tissues was determined using immunohistochemistry (IHC) analysis. (c) Low A2ML1 protein expression in ESCC tissues was determined using IHC analysis. (d) A2ML1 protein levels in 94 ESCC and normal tissues were analyzed; ^*∗∗∗∗*^*P* < 0.0001, Student's *t*-test. (e) Statistical analysis of the association between A2ML1 expression and survival probability in patients with ESCC; ^*∗∗∗∗*^*P* < 0.0001, KMPLOT survival analysis.

We first analyzed the expression of A2ML1 in EC using the TCGA (https://cancergenome.nih.gov/) and GTEx databases (https://gtexportal.org/home/). Open data showed that the level of A2ML1 in EC tissues was significantly lower than that in normal esophageal tissues (Figure 1(a); *P* < 0.01). To validate this finding, we analyzed the correlation between A2ML1 expression and clinicopathological parameters in 94 patients with ESCC. Similarly, the protein level of A2ML1 in tumor tissues (Figures 1(b)–1(d); *P* < 0.0001) was significantly lower than that in normal tissues. Moreover, high A2ML1 expression was correlated with tumor size (Table 1; *P* < 0.01). Cox analysis showed that low A2ML1 expression was an independent risk factor associated with poor prognosis in patients with ESCC (Table 2; *P* < 0.0001). KMPLOT survival analysis also revealed that low A2ML1 expression was significantly associated with poor prognosis (Figure 1(e); hazard ratio (HR) = 0.294, 95% confidence interval (CI) = 0.176–0.492, and *P* < 0.0001).

### 3.2. A2ML1 Functioned as a Tumor Suppressor in ESCC

**Figure 2:**
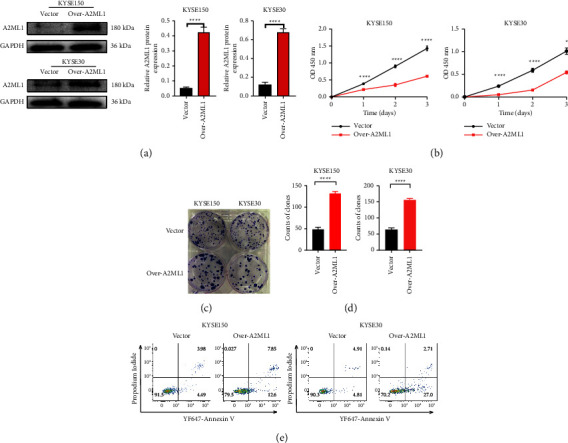
A2ML1 inhibited ESCC cell proliferation and promoted apoptosis. (a) Western blot showing A2ML1 expression in KYSE150 and KYSE30 cells transfected with A2ML1 plasmid; ^*∗∗∗∗*^*P* < 0.0001, Student's *t*-test. (b) The Cell Counting Kit-8 assay revealed the reduced proliferative ability of KYSE150 and KYSE30 cells after transfection; ^*∗*^*P* < 0.05, ^*∗∗∗∗*^*P* < 0.0001, Student's *t*-test. (c) Colony formation assay of A2ML1-overexpressed KYSE150 and KYSE30 cells. (d) Quantitative analysis of clones in each group; ^*∗∗∗∗*^*P* < 0.0001, Student's *t*-test. (e) Yellow fluorescent 647-conjugated Annexin V/propidium iodide double staining was used to assess KYSE150 and KYSE30 cell apoptosis after transfection with the A2ML1 plasmid.

To assess the function of A2ML1 in ESCC, we increased its expression in KYSE30 and KYSE150 cell lines through transfection with the A2ML1 plasmid. Immunoblotting confirmed the presence of A2ML1 in both KYSE30 and KYSE150 cell lines (Figure 2(a)). The CCK-8 assay results showed that transfection with A2ML1 significantly inhibited the proliferation of KYSE30 and KYSE150 cells (Figure 2(b); *P* < 0.05 and *P* < 0.0001, respectively). This inhibitory effect was consistently observed in the impaired colony formation ability of both cell lines (Figures 2(c)–2(d); *P* < 0.0001). ESCC cell apoptosis was measured using YF647-Annexin V and PI staining. Flow cytometry results showed a significant difference in the apoptosis rate between the vector- and A2ML1 plasmid-transfected cells (Figure 2(e)). These findings suggest that A2ML1 inhibited the proliferation and promoted the apoptosis of ESCC cells.

### 3.3. A2ML1 Exerted an Antiproliferative Effect in ESCC *In Vivo*

**Figure 3:**
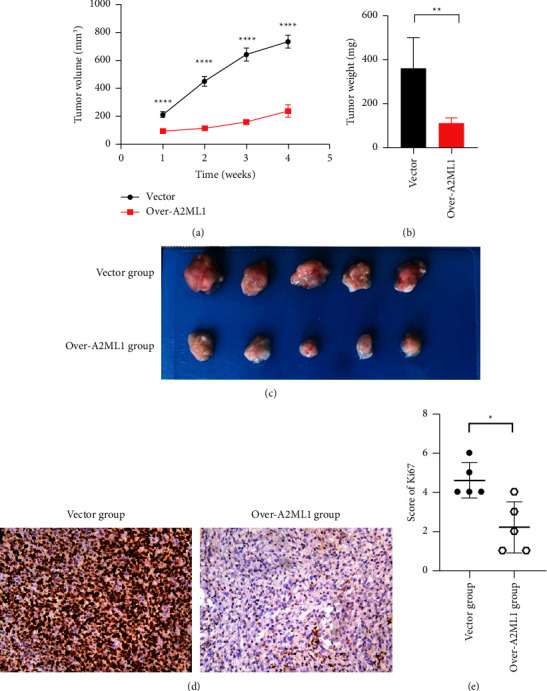
A2ML1 overexpression suppressed ESCC growth *in vivo*. (a) Subcutaneous tumor growth was monitored weekly after ESCC cell injection according to the tumor volume. (b) Four weeks after the tumor cell injection, the tumor nodes were isolated and weighed to compare their differences; ^*∗∗∗∗*^*P* < 0.0001, Student's *t*-test. (c) Representative images of tumor nodes; ^*∗∗*^*P* < 0.01, Student's *t*-test. (d) Representative images of Ki67 protein expression in the vector and A2ML1 overexpression groups. (e) IHC staining for Ki67 in mouse tumor tissue from the vector and overexpression groups and scatter plot for Ki67 expression; ^*∗*^*P* < 0.05, Student's *t*-test.

To determine the role of A2ML1 in inhibiting ESCC proliferation *in vivo*, we treated KYSE150 cells with a lentivirus containing the A2ML1 plasmid and subcutaneously injected the cells into mice. Compared to the control group, the tumors derived from A2ML1 overexpression ESCC cells were smaller and the growth rate was lower (Figure 3(a), *P* < 0.0001; 3(b), *P* < 0.01; 3(c)). IHC staining further confirmed that the proliferation index of Ki67 protein in the tumor tissue was significantly lower than that in the control group (Figures 3(d)−3(e)). These results show that the overexpression of A2ML1 inhibited ESCC cell growth *in vivo*.

### 3.4. p21 Protein Expression Increased in ESCC Cells with A2ML1 Overexpression

**Figure 4:**
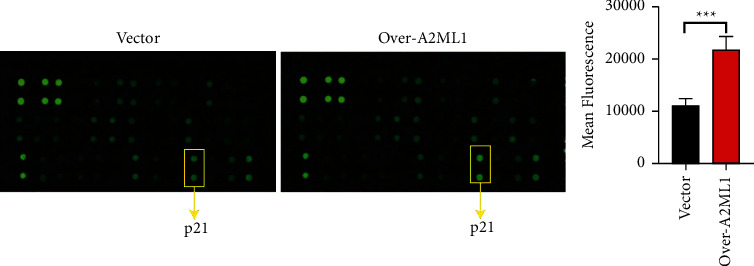
Screening of apoptosis factors associated with A2ML1 overexpression in KYSE150 cells; p21-specific data are indicated. ^*∗∗∗*^*P* < 0.001, Student's *t*-test.

In view of the correlation between A2ML1 expression level and tumor size, as well as the experimental finding that A2ML1 overexpression inhibits ESCC cell proliferation and promotes apoptosis, we speculated that A2ML1 may participate in tumor cell growth regulation. Therefore, we used a human apoptosis antibody kit to identify proteins associated with cell proliferation and apoptosis. The results showed increased p21 protein expression in the A2ML1 overexpression group. Array data related to p21 are shown in yellow boxes (Figure 4). These results indicate that A2ML1 might inhibit tumor growth by impeding cell proliferation.

## 4. Discussion

In this study, we observed a significant decrease in A2ML1 protein expression in ESCC tissues compared to that in adjacent normal tissues, as evidenced by IHC staining. Higher levels of A2ML1 protein were correlated with smaller ESCC tumor size and improved tumor-specific survival rates. Furthermore, multivariate analysis established A2ML1 as a novel independent prognostic factor for ESCC. A2ML1 overexpression significantly inhibited proliferation and colony formation and promoted apoptosis in KYSE30 and KYSE150 cells. Similarly, A2ML1 played a role in tumor inhibition in mouse models. The apoptosis microarray results revealed a decrease in the expression of the apoptosis-inhibiting protein survivin along with an increase in the proliferation-inhibiting protein p21 downstream of the ESCC cells overexpressing A2ML1. These results suggest that A2ML1 inhibited ESCC cell proliferation and promoted apoptosis.

A2ML1 has demonstrated clinical significance in paraneoplastic pemphigus (PNP), an autoimmune bullous disease characterized by pleomorphic mucosal skin lesions, mainly associated with lymphoproliferative tumors. Patients with PNP exhibit clinically severe mucosal skin lesions similar to conditions such as pemphigus vulgaris, erythema multiforme, and lichen planus. Related tumors include non-Hodgkin's lymphoma, chronic lymphocytic leukemia, Castleman's disease, and thymoma [15–17]. Moreover, A2ML1 has proven effective as a diagnostic biomarker for PNP [18, 19]. A bioinformatics study has reported the potential of A2ML1 in predicting cancer prognosis, with its expression linked to the tripartite motif containing 58/cg26157385 methylation [12]. Previous studies based on the comprehensive analysis of genomics, epigenomics, transcriptomics, and clinical information of pancreatic cancer have demonstrated a significant negative correlation between *A2ML1* gene expression level and methylation status. This led to the speculation that methylation status played an important role in gene expression and prognosis. These results suggest that A2ML1 may be a potential novel biomarker for the treatment, diagnosis, and prognosis of pancreatic cancer [13].

The detection of apoptosis-related proteins using a human apoptotic antibody kit showed that the expression of the apoptosis-inhibiting protein survivin and proliferation-inhibiting protein p21 were different between A2ML1 overexpression ESCC cells and the control group. p21, a well-established cyclin-dependent kinase inhibitor, plays an important role in controlling cell-cycle progression [20, 21]. Moreover, p21 is mainly associated with p53 during cell-cycle arrest. Studies have demonstrated the existence of p53-independent pathways leading to p21 induction in the early years of p21 discovery [22]. p21 has also been associated with the sensitivity of cells to transforming growth factor-*β* [23, 24]. However, the upstream and downstream mechanisms of A2ML1 in tumors require a systematic investigation using high-throughput techniques, such as RNA sequencing and mass spectrometry.

## 5. Conclusions

Our results revealed a correlation between low A2ML1 expression and poor prognosis in ESCC. Moreover, both clinical samples and experimental studies corroborated the inhibitory effect of A2ML1 on ESCC progression. The expression of A2ML1 in ESCC tissues holds promise as an independent prognostic factor, providing new insights into its role in tumorigenesis. However, the specific molecular mechanisms of A2ML1 in ESCC cells require further exploration.

## Figures and Tables

**Table 1:** Association between A2ML1 protein expression and clinicopathological factors.

Factors	Cases (*n* = 94)	A2ML1 protein expression		*P* value
		Low (*n* = 48, %)	High (*n* = 46, %)	
*Age*				
<60 years	19	10 (20.83)	9 (19.57)	0.8784
≥60 years	75	38 (79.17)	37 (80.43)	

*Sex*				
Female	28	15 (31.25)	13 (28.26)	0.7514
Male	66	33 (68.75)	33 (71.74)	

*T stage*				
T1-2	41	13 (31.25)	28 (28.26)	0.0010^*∗∗∗*^
T3-4	53	35 (68.75)	18 (71.74)	

*N stage*				
N0	57	31 (64.58)	26 (56.52)	0.4239
N+	37	17 (35.42)	20 (43.48)	

*Differentiation*				
Well	58	28 (58.33)	30 (65.22)	0.4925
Moderate-poor	36	20 (41.67)	16 (34.78)	

*Distant metastasis*				
M0	94	48 (100)	46 (100)	
M1	0	0 (0)	0 (0)	

^*∗∗∗*^ *P* < 0.001, chi-square test. A2ML1, *α*2-macroglobulin-like 1.

**Table 2:** Cox proportional hazard regression analyses for overall survival.

Factors	Univariate analysis		Multivariate analysis	
	HR (95% CI)	*P* value	HR (95% CI)	*P* value
*Age*				
<60 years				
≥60 years	1.045 (0.591–1.848)	0.8808		

*Sex*				
Female				
Male	0.848 (0.512–1.404)	0.5217		

*T stage*				
T1-2				
T3-4	1.984 (1.222–3.221)	0.0056^*∗∗*^	1.376 (0.819–2.312)	0.2285

*N stage*				
N0				
N+	1.42 (0.888–2.271)	0.1434	1.377 (0.836–2.27)	0.209

*Differentiation*				
Well				
Moderate-poor	1.377 (0.837–2.264)	0.2076	1.349 (0.795–2.288)	0.2669

*A2ML1 protein level*				
Low				
High	0.232 (0.137–0.392)	<0.0001^*∗∗∗∗*^	0.252 (0.146–0.436)	<0.0001^*∗∗∗∗*^

^*∗∗*^ *P* < 0.01; ^*∗∗∗∗*^*P* < 0.0001; A2ML1, *α*2-macroglobulin-like 1; CI, confidence interval; HR, hazard ratio.

## Data Availability

The data used to support the findings of this study are included within the article.
